# Ice-regenerated flame retardant and robust film of *Bombyx mori* silk fibroin and POSS nano-cages†

**DOI:** 10.1039/c7ra13708g

**Published:** 2018-02-28

**Authors:** Luca Valentini, Silvia Bittolo Bon, Nicola M. Pugno

**Affiliations:** Dipartimento di Ingegneria Civile e Ambientale, Università di Perugia, UdR INSTM Strada di Pentima 4 05100 Terni Italy luca.valentini@unipg.it +39 0744 492924.; Laboratory of Bio-Inspired and Graphene Nanomechanics, Department of Civil, Environmental and Mechanical Engineering, University of Trento Via Mesiano 77 Trento Italy nicola.pugno@unitn.it +39 0461 282525; School of Engineering and Materials Science, Queen Mary University of London Mile End Road London E14NS UK; Ket-Lab, Edoardo Amaldi Foundation, Italian Space Agency via del Politecnico snc I-00133 Roma Italy

## Abstract

In this study, we present a simple method to prepare and control the structure of regenerated hybrid silkworm silk films through icing. A regenerated hybrid silk (RHS) film consisting of a micro-fibrillar structure was obtained by partially dissolving amino-functionalized polyhedral oligomeric silsesquioxanes (POSS) and silk fibers in a CaCl_2_–formic acid solution. After immersion in water and icing, the obtained films of RHS showed polymorphic and strain-stiffening behaviors with mechanical properties that were better than those observed in dry or wet-regenerated silk. It was also found that POSS endowed the burning regenerated silk film with anti-dripping properties. The higher β-sheet content observed in the ice-regenerated hybrid micro-fibrils indicates a useful route to fabricate regenerated silk with physical and functional properties, *i.e.* strain-stiffening, similar to those observed to date in natural spider silk counterpart and synthetic rubbers, and anti-dripping of the flaming melt. Related carbon nanotube composites are considered for comparison.

## Abstract 2

In this study, we present a simple method to prepare and control the structure of regenerated hybrid silkworm silk films through icing.

## Graphical abstract

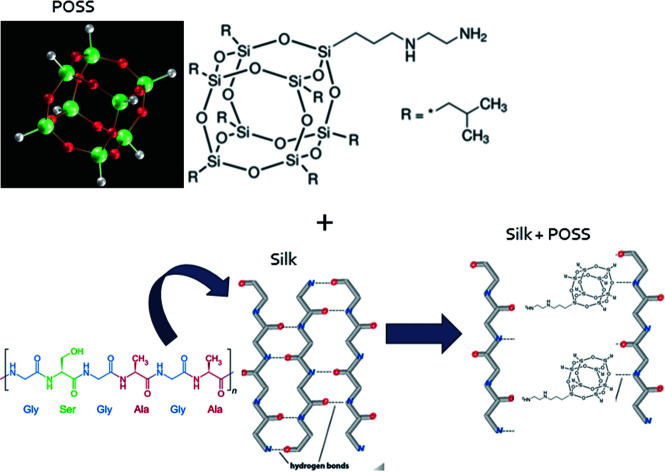

## Introduction

Silk is a fascinating natural material that combines exceptional mechanical properties in terms of strength, stiffness, and resilience;^1,2^ therefore, there is an increasing interest to emulate the properties of natural silk by mimicking the natural process in regenerated silk.^3–5^

It is generally believed that the exceptional mechanical properties of silk originate from the combination of a hierarchical architecture of the β-sheet crystal and fibrillar structures. Several attempts have been made in this regard. Hu *et al.*,^6^ for example, were able to regulate the crystallinity of the regenerated silk fibroin by exposing silk to hot water vapor; Zhang *et al.*^7^ showed the preparation of silk directly by dissolving the fibers in CaCl_2_–formic acid, preserving the nano-fibril structure, and allowing high-quality silk materials; on the other hand, recently, Buehler *et al.*^8^ reported a bioinspired spinning method to obtain regenerated silk fibers, with interesting mechanical properties, by pulling out a silk microfibril solution.

However, the methods adopted to reassemble silk fibroin in thin films resulted in films that became brittle once dried or had low strength in the wet state.^9,10^ A recent study demonstrates that the partial dissolution of silk fibers can be the hidden ingredient to obtain hierarchical micro-fibrils with a high content of β-sheet crystals.^8^ Recent advances on the structure of regenerated liquid silk fibroin help us gain a deeper understanding of the effect of fiber dissolution on the properties of silk fibroin and provide important experimental data for using silk protein as advanced functional biomaterials.^11–13^

Polyhedral oligomeric silsesquioxanes (POSS) are organic–inorganic molecules, approximately 1–3 nm in diameter, with the general formula (RSiO_1.5_)_*n*_, where R is hydrogen or a functional group.^14–16^ The incorporation of amino-functionalized POSS molecules (*i.e.* NH_2_-terminated POSS) into silk could lead to the dispersion of these nano-cages through the coupling of the amino groups and the oxygen atoms within the dissolved silk chains. Moreover, POSS is a low-cost material that is generally used in synergy with the polymer phase as a stiff phase with the Young's modulus reaching 7.5 GPa;^17^ in addition, it is usually used as a flame-retardant in polymer nanocomposites due to the retention of the silicon phase during combustion; this leads to the reduction of flammability and the formation of a glassy char acting as a barrier to heat.^18^

Herein, we report a study that exploits the ability of polyhedral oligomeric silsesquioxanes to promote the partial dissolution of silk fibers and act as a reinforcing agent. The post-synthesis icing has been used to regulate the β-sheet crystal content during the crystallization process of these hybrid micro-fibrils. We have observed that the ice-regenerated hybrid silk film displays better mechanical performances and a strain-stiffening character that can be regulated by the β-sheet crystal content and a reduced flammability.

## Experimental

For the preparation of the regenerated hybrid silk film, commercial *B. mori* silk cocoons were boiled for 1 h in a distilled water solution of 0.025 wt% NaHCO_3_ and then rinsed with distilled water every 30 min to remove sericin. According to the method adopted by Kaplan *et al.*,^19^ the degummed silk (*i.e.* 0.2 g) and aminopropyl heptaisobutyl polyhedral oligomeric silsesquioxanes (*i.e.* 2 mg) (hereinafter named amino-functionalized POSS were purchased from Hybrid Plastics (USA) as a crystalline powder and used as received) were then added to a CaCl_2_ (*i.e.* 0.14 g)–formic acid (*i.e.* 20 ml) solution and stirred overnight at 40 °C yielding a 1 wt% solution. The same procedure was adopted by introducing short-COOH functionalized multi-walled carbon nanotubes (CNTs) supplied by Cheaptubes (outer diameter: 20–30 nm, inside diameter: 5–10 nm, purity: >95 wt%, length: 0.5–2.0 μm). Regenerated hybrid silk films were prepared by leaving the silk-amino-functionalized POSS and silk-functionalized CNT solution to evaporate for 12 h in a polystyrene Petri dish (diameter 15 cm). The resulting RHS and RS–CNT films were then immersed in distilled water for 5 min and subsequently frozen for 3 h at −10 °C. Both the water-annealed and iced films were air-dried at 25 °C before characterization. The morphology of the films was investigated by optical and field emission scanning electron microscopy (FESEM). Fourier transform infrared (FTIR) analysis was performed using a Jasco FTIR FT/IR-615 spectrometer in the ATR mode in the wavenumber range from 400 to 4000 cm^−1^. Differential scanning calorimetry (DSC) was carried out using TA Q200. Each sample was heated from 25 °C to 214 °C at the heating rate of 10°C min^−1^; the samples were then cooled down to room temperature and heated again. Thermogravimetric analysis (TGA) was performed using SII TG/DTA 6300 (Seiko). The samples were heated from room temperature to 800 °C at the heating rate of 10°C min^−1^. The rate of gas (air or nitrogen) was 70 ml min^−1^.

X-ray diffraction was performed using the Bruker D8 Advance diffractometer, with a CuKα radiation source and wavelength *λ* = 0.154 nm, operated at 40 kV and 40 mA. The incidence angle (2*θ*) was varied between 2° and 60°, and the scan rate was 0.02° s^−1^.

The tensile properties, *i.e.* toughness, Young's modulus, and tensile strength, of the films were measured using a universal tensile testing machine (Lloyd Instr. LR30K) with a 50 N static load cell. The film samples were cut into strips (30 mm × 12 mm). The gauge length was 20 mm, and the extension rate was set at 1 mm min^−1^.

## Results and discussion

**Fig. 1:**
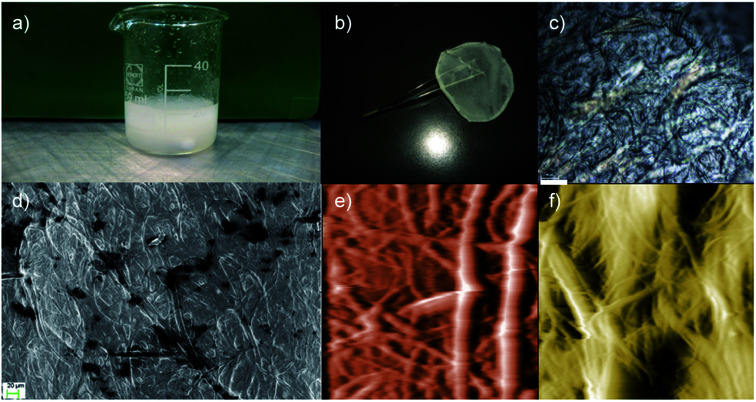
Visual appearance and structural characterization of the regenerated hybrid silk. (a) Visual appearance of the POSS/Silk/CaCl_2_ mixture, (b) RHS film, (c) polarized light microscopy image of the RHS film, and (d) FESEM image of the RHS film. After 24 hours, the AFM images (70 μm × 70 μm) show that the silk fiber in the (e) CaCl_2_–formic acid and (f) POSS/CaCl_2_–formic acid solution partially dissolved into micro fibrils with diameters of about 10 μm and 5 μm, respectively. False color is used in the AFM images. Scale bars are 60 μm in (c) and 20 μm in (d).

The outstanding mechanical properties of the natural silk originate from its intrinsic hierarchical order. Previous studies have indicated the importance of the complete dissolution of silk in several solvents that destroys the hierarchy once the films are dried.^20–24^ Herein, we used the CaCl_2_–formic acid dissolution system and introduced the amino-functionalized POSS (Fig. 1a–f). This new approach results in a uniform viscous solution (Fig. 1a) that produces films (Fig. 1b) with a micro-fibrillar structure (Fig. 1c–f). Interestingly, the RHS film presents light diffraction (Fig. 1c) typical of nematic liquid-crystal configuration. These viscous liquid crystals allow the orientation of the micro-fibrils along the drawing direction, as shown below. The AFM and FESEM analyses on silk fibers dissoluted in the POSS CaCl_2_–formic acid solution show how the silk disaggregates from macro fibers into a micro-fibril structure.

**Fig. 2:**
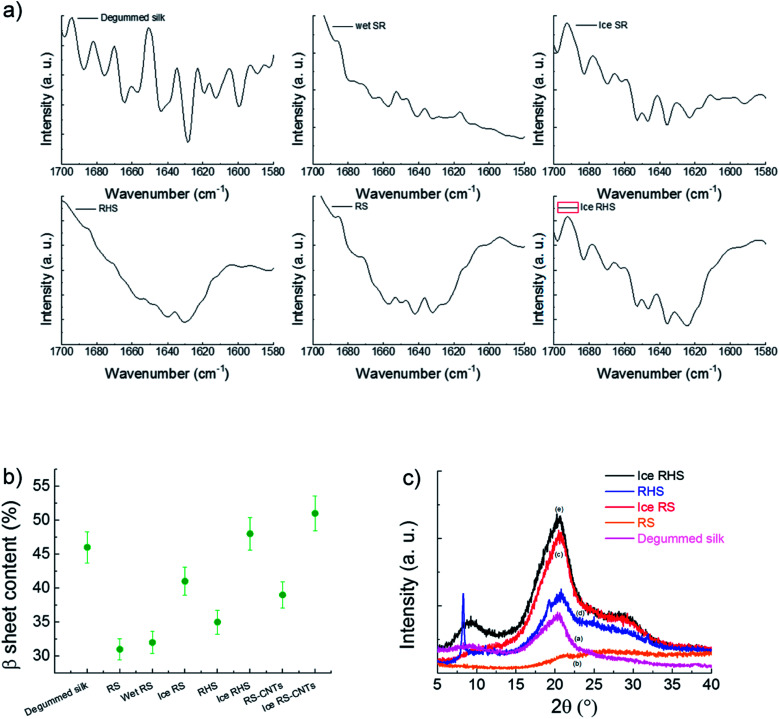
(a) FTIR spectra of the degummed silk, regenerated silk after water annealing (wet RS), regenerated silk after icing (ice RS), regenerated hybrid silk (RHS), regenerated silk (RS), and regenerated hybrid silk after icing (ice RHS). (b) Calculated crystallinity of the prepared samples. (c) XRD results of the degummed silk (a), RS (b), ice RS (c), RHS (d), and ice RHS (e) films.

FTIR characterization was used to estimate the β-sheet (crystalline) content (Fig. 2a). Deconvolution of the amide I region (1580–1700 cm^−1^) was performed *via* the Origin 9.0 software by smoothing the amide I region with a nine-point Savitzky–Golay smoothing filter, whereas deconvolution was performed using Lorentzian line shape (see Fig. S1†). The ratio between the peak area in the wavenumber region of 1600–1640 cm^−1^, which is the main absorbance region of the β-sheet crystal in amide I,^25^ and the whole area of the amide I has been used to estimate the β-sheet content. The deconvolution of the amide I band^25^ provides an estimation of 31 ± 3%, 35 ± 3%, and 48 ± 3% content of the β-sheet structure in the RS, RHS, and ice RHS, respectively, and the β-sheet structure content of the degummed *B. mori* silkworm silk is 46 ± 2% (Fig. 2b). The β-sheet content in the RS–CNTs and ice RHS–CNTs was found to be 39 ± 3% and 51 ± 3%, respectively (see Fig. S1†). Changes in the structure of the silk films prepared *via* various treatments were also investigated by XRD analysis. In previous studies, three silk fibroin conformations have been identified by X-ray diffraction: random coil, silk I, and silk II.^26^Fig. 2c shows the XRD data for the degummed silk, RS, ice RS, RHS, and ice RHS films. The degummed silk film showed the II silk structure,^7^ whereas the RS film exhibited an amorphous state, characterized by the presence of a broad peak in the 2*θ* scattering angle range from 5° to 40°. The ice RS film exhibited the typical X-ray diffractogram of the silk I structure, having diffraction peaks at 20.4° and 29.4°.^27^ The ice RHS film was characterized by the diffraction peaks at the 2*θ* values of 9°, 20.4°, and 29.4°, corresponding to the silk I and silk II structures. Compared with that of the RHS film, the silk II peak at 9° disappeared, and the POSS peak at 8.3° appeared.^28^ The results indicate that both ice and POSS addition have a significant influence on the formation of the silk I structure.

**Fig. 3:**
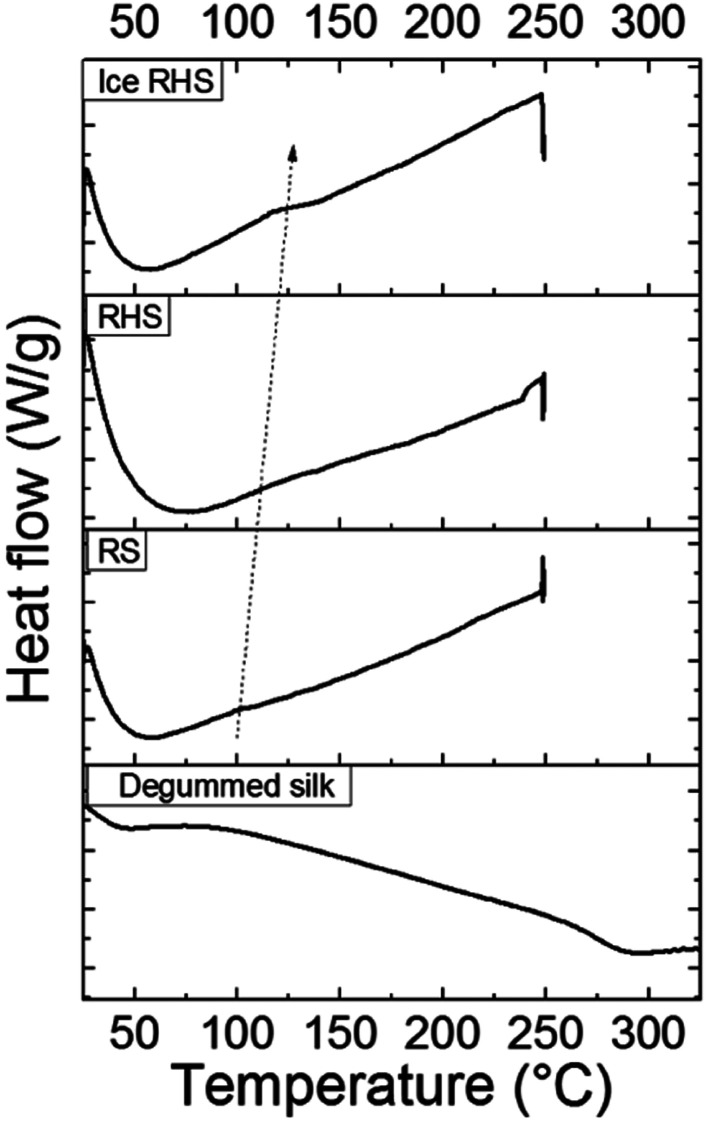
DSC curves for the regenerated silk (RS), regenerated hybrid silk (RHS), and ice regenerated hybrid silk (ice RHS). Degummed silk was used as a control.

Fig. 3 shows the DSC curves for RS, wet RS, ice RS, RHS, and ice RHS; degummed silk is used as a control. RS, RHS, and RHS after icing showed a small endothermic step below 170 °C, and a degradation peak at about 250 °C thereinafter. The endothermic step below 170 °C was due to glass transitions of the samples, and its intensity increased with an increase in the crystallinity of the films (see Fig. 2b).^6^ After the appearance of glass transition, no other peaks before thermal degradation were detected; this implied that the β-sheet crystals were formed during the ice treatment and the thermal energy during the DSC scan did not induce a significant increment of the β-sheet content.

**Fig. 4:**
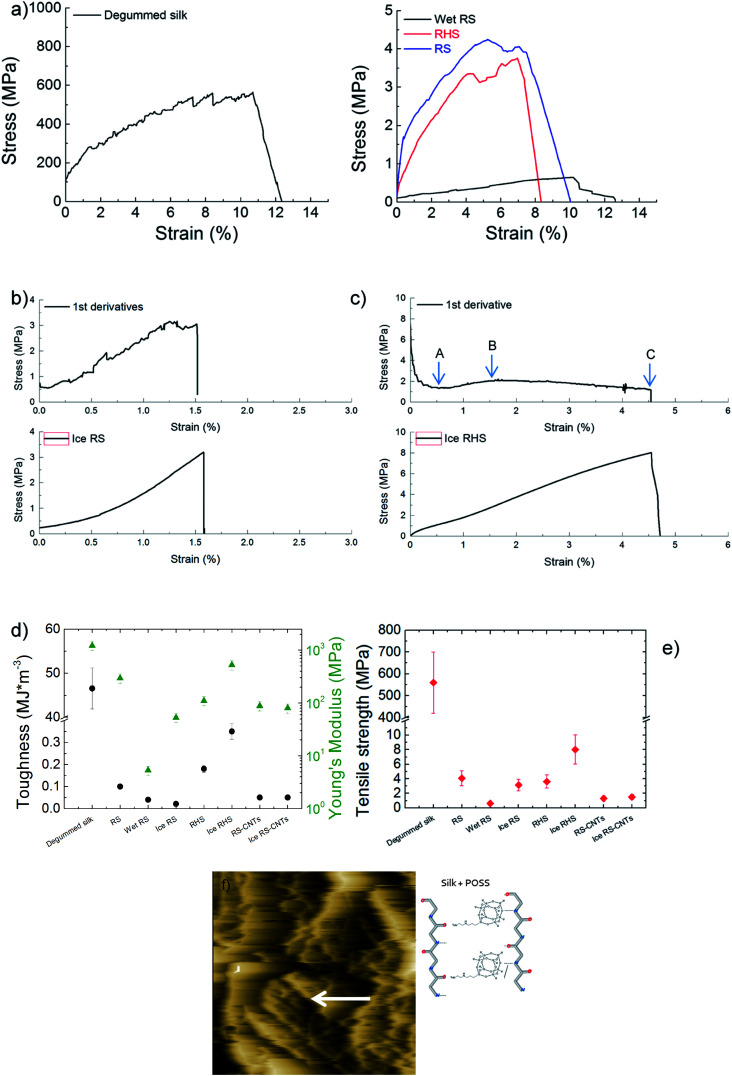
(a) Tensile stress–strain average curves of the degummed silk, RS film after water annealing, and RHS and RS films (5 samples averaged). (b and c) Tensile stress–strain curves of (b) RS and (c) RHS films after icing; the curves in the top panels are the first derivative lines of the stress–strain curves reported in the bottom panels (solid black lines, 5 samples averaged). The strain-stiffening strain ratio is the strain-stiffening-induced-strain (the strain from point A to point B) over the total strain of the non-linear region (from point A to the elongation break C). (d) Toughness (circles) and Young's modulus (triangles), and (e) tensile strength of the samples. (f) Cross-sectional AFM image of the ice RHS after tensile fracture, which reveals micro fibrils strained along the drawing direction indicated by the white arrow. The schematic of the proposed mechanism of POSS for providing additional bonding sites between the silk chains is reported.

The mechanical characterizations of the degummed silk, regenerated silk, wet-regenerated silk, and regenerated hybrid silk (Fig. 4a) show that all these display a yield point followed by the so-called strain-softening behaviour where the slope of the stress–strain curves decreases with strain. In particular, after losing the hierarchical structure of the degummed natural silk, RS results in a material with poor crystalline fraction and thus with scarce mechanical properties. Moreover, since the RS film is composed of silk micro-fibrils and Ca ions, its mechanics can be tuned by water annealing.^7^ Hence, calcium ion captures the water molecules, which acts as plasticizers, resulting in soft and stretchable RS films. Thus, wet-regenerated silk films showed a lower Young's modulus and yield point and a higher elongation due to the plasticization effect of water.^19^ Although immersion in water helps in the removal of both CaCl_2_ and formic acid and induces the β-sheet reconstruction, the RS films become more brittle in the dry state (Fig. 4a) as compared to the wet samples. On the contrary, after the yield point, the RS and RHS after icing (Fig. 4b–c) show a strain-stiffening behaviour with the slope of the stress–strain curves that thus increases with strain. This strain-stiffening occurs in spider silk dragline^29,30^ where first the intra-molecule β-sheet unfolds (region between A and B in Fig. 4c), and then, the tensile deformation causes breaking of the crystallites; this gives rise to a strain-softening region (region between B and C in Fig. 4c). Fig. 4d and e summarize the trend of toughness, Young's modulus, and tensile strength of the tested samples. The tensile strength of the RS film is 4.3 ± 0.9 MPa and the toughness is 0.10 ± 0.02 MJ m^−3^; the toughness of the RHS film is improved to 0.20 ± 0.02 MJ m^−3^ with a tensile strength of 4.0 ± 0.8 MPa. After icing, the tensile strength and toughness of the RHS film improved to 8.0 ± 1.6 MPa and 0.35 ± 0.07 MJ m^−3^, respectively. Thus, the RHS film after icing shows highest mechanical properties, demonstrating a stress–strain behaviour similar in shape to that of spider silk. From the cross-section fracture morphology of the RHS film after icing (Fig. 4f), it is clear that the silk micro-fibrils show pull-out. This finding can support a crack propagation model where the fracture mechanism is activated in the strain-stiffening region by breaking of the bonds between the amino-functionalized POSS nano-cages and the silk chains that are stretched at the same time; this results in the dissipation of mechanical energy. Finally, ice-regenerated hybrid silk film shows a strain-stiffening strain ratio, defined as the strain-stiffening-induced strain over the total strain of the non-linear region, that is about 35%.

**Fig. 5:**
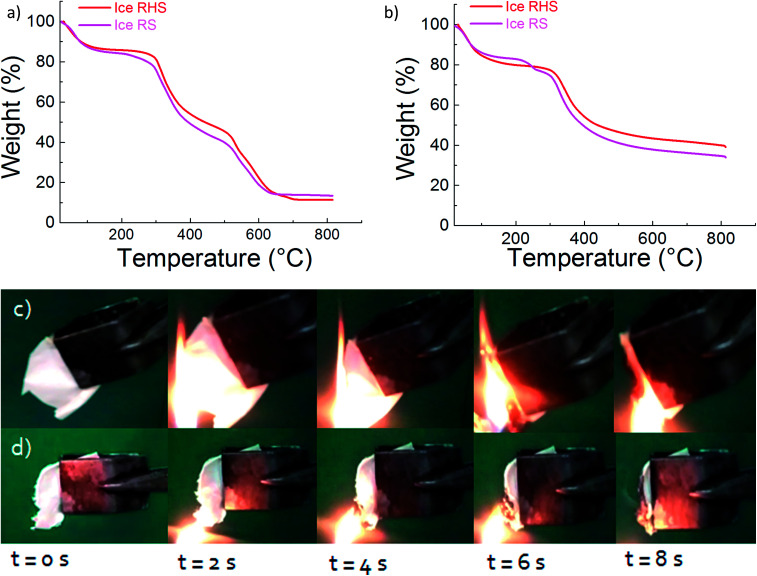
Residual weight in percentage *vs.* temperature for ice RS and ice RHS films, with a heating rate of 10°C min^−1^ under an (a) air atmosphere and under (b) a nitrogen atmosphere. Combustion process of (c) ice RS and (d) ice RHS films. The introduction of POSS into RS extinguishes the flame and causes anti-dripping during the combustion process.

Fig. 5 shows the TGA results for the ice RS and ice RHS films under both air and nitrogen atmosphere. Under the air atmosphere (Fig. 5a), the addition of POSS improved the thermal stability of the RS significantly at above 200 °C. The final decomposition temperature increased from 610 °C to 700 °C, with the incorporation of POSS. Under a nitrogen atmosphere (Fig. 5b), the char yield increased up to 43% with the addition of POSS.

Flame self-extinguishing is a desirable property for silk films mainly if these films are used in silk textiles. Previous studies demonstrated that chemically modified silk fibroin fibers retained a high level of flame retardancy.^31^ However, the flame retardant finishing technology for silk is still challenging. Fig. 5 shows the burning behavior of the RS and RHS films after the ice treatment. In the case of ice RS and ice RS–CNTs (Fig. 5c and S3†), the samples ignited instantly with the flame extended to the entire sample with severe dripping of the flaming melt. For the ice RHS film (Fig. 5d), the ignition behavior was found to be similar to that of the ice RS film; however, the flame vanished with char formation. Since the burning process is reduced by the formation of glassy char of POSS, it can be said that most of the POSS molecules are dispersed homogeneously into the silk chains. Hence, an adequate content of the POSS hybrid silk film can generate char to prevent the flame from spreading.

## Conclusions

In summary, a regenerated hybrid silk film was fabricated by the solvent casting approach. We found that the addition of amino-functionalized POSS to the CaCl_2_–formic acid/silk mixture induced partial dissolution of natural fibers with a micro-fibrillar structure. The attractive characteristic of these films made of regenerated hybrid silk include the possibility to tune the crystalline content *via* the icing method. After icing, these films displayed a higher crystalline fraction and a higher toughness as compared to the non-hybrid regenerated counterpart. Finally, the strain-stiffening behaviour as well as anti-dripping of the flaming melt were observed on the ice regenerated simple and hybrid silk. This simple and facile fabrication method can be further exploited for incorporating different functional inorganic species to obtain thin films with unique properties.

## Conflicts of interest

There are no conflicts of interest to declare.

## Supplementary Material

RA-008-C7RA13708G-s001
