# Supplementary material for: Ice-regenerated flame retardant and robust film of Bombyx mori silk fibroin and POSS nano-cages
Source: RSC Adv. 2018 Feb 28;8(17):9063–9. doi: 10.1039/c7ra13708g (PMC9078597; doi:10.1039/c7ra13708g)
Supplement: RA-008-C7RA13708G-s001 [file RA-008-C7RA13708G-s001.pdf]

Electronic Supplementary Material (ESI) for RSC Advances.
This journal is © The Royal Society of Chemistry 2018

Supplementary data

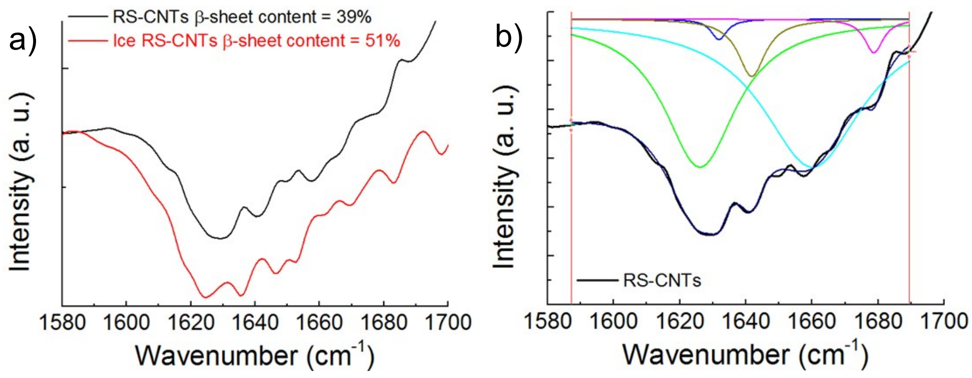

Figure S1. (a) Transmittance spectra of RS-CNTs and icing RS-CNTs films, amide I, obtained after (b) Fourier deconvolution. The heavy line represents the deduced transmittance band.

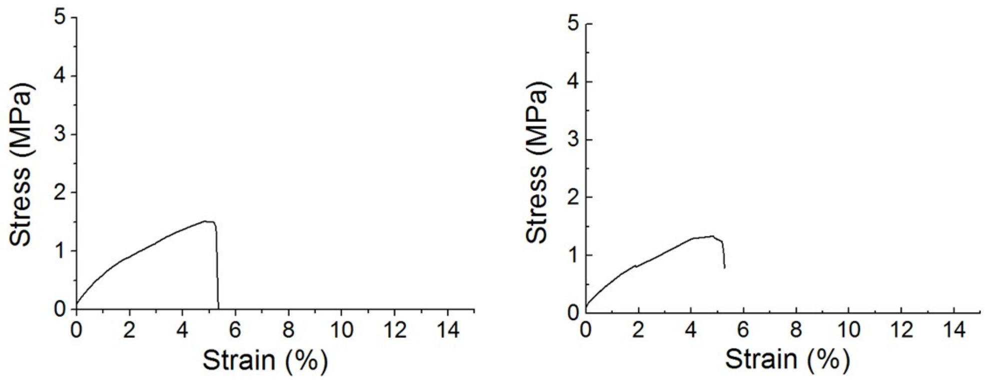

Figure S2. Tensile stress-strain average curves of (a) RS-CNT and (b) RS-CNTs films after icing.

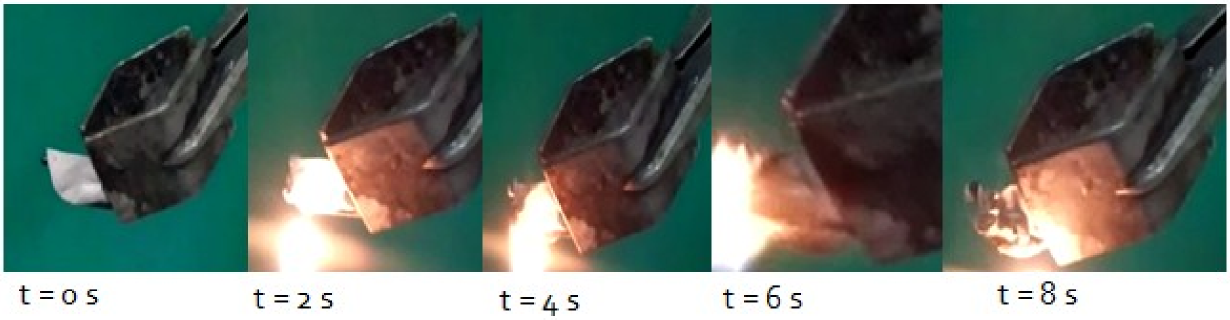

Figure S3. Combustion process of ice RS-CNTs film. The introducing of CNTs into RS enhances the flame propagation and produces severe dripping during the combustion process.
